# Revealing the Timeline of Structural MRI Changes in Premanifest to Manifest Huntington Disease

**DOI:** 10.1212/NXG.0000000000000617

**Published:** 2021-10-12

**Authors:** Peter A. Wijeratne, Sara Garbarino, Sarah Gregory, Eileanoir B. Johnson, Rachael I. Scahill, Jane S. Paulsen, Sarah J. Tabrizi, Marco Lorenzi, Daniel C. Alexander

**Affiliations:** From the Centre for Medical Image Computing (P.A.W., D.C.A.), Department of Computer Science, University College London, Gower Street; Huntington's Disease Research Centre (P.A.W., S. Gregory, E.B.J., R.I.S., S.J.T.), Department of Neurodegenerative Disease, University College London, Queen Square Institute of Neurology, London, United Kingdom; Dipartimento di Matematica (S. Garbarino), UNIGE, DIMA, Genova, Italy; Departments of Neurology and Psychiatry (J.S.P.), Carver College of Medicine, University of Iowa; and Université Côte d’Azur (M.L.), Inria, Epione Research Project, Valbonne, France.

## Abstract

**Background and Objectives:**

Longitudinal measurements of brain atrophy using structural MRI (sMRI) can provide powerful markers for tracking disease progression in neurodegenerative diseases. In this study, we use a disease progression model to learn individual-level disease times and hence reveal a new timeline of sMRI changes in Huntington disease (HD).

**Methods:**

We use data from the 2 largest cohort imaging studies in HD—284 participants from TRACK-HD (100 control, 104 premanifest, and 80 manifest) and 159 participants from PREDICT-HD (36 control and 128 premanifest)—to train and test the model. We longitudinally register T1-weighted sMRI scans from 3 consecutive time points to reduce intraindividual variability and calculate regional brain volumes using an automated segmentation tool with rigorous manual quality control.

**Results:**

Our model reveals, for the first time, the relative magnitude and timescale of subcortical and cortical atrophy changes in HD. We find that the largest (∼20% average change in magnitude) and earliest (∼2 years before average abnormality) changes occur in the subcortex (pallidum, putamen, and caudate), followed by a cascade of changes across other subcortical and cortical regions over a period of ∼11 years. We also show that sMRI, when combined with our disease progression model, provides improved prediction of onset over the current best method (root mean square error = 4.5 years and maximum error = 7.9 years vs root mean square error = 6.6 years and maximum error = 18.2 years).

**Discussion:**

Our findings support the use of disease progression modeling to reveal new information from sMRI, which can potentially inform imaging marker selection for clinical trials.

Identification of new biomarkers of disease progression is crucial for the efficient design and execution of clinical trials in Huntington disease (HD),^1^ and more broadly any neurodegenerative disease. Structural MRI (sMRI) measured at more than 1 time point can provide continuous measures that track disease progression (i.e., biomarkers), and methods such as voxel-based morphometry can be used to directly estimate longitudinal changes in sMRI data.^2^ Direct regression models (i.e., those that do not account for hidden variables) have also been used to model dynamic change in sMRI measures in Alzheimer disease (AD),^3^ Parkinson disease,^4^ and HD.^5^ However, longitudinal analysis in medical data is confounded by intersubject variability, measurement noise, and the lack of a common reference timeline, as study participants are typically drawn from a mixture of unknown or broadly defined disease times. This makes it difficult to establish a common disease timeline and hence identify suitable markers of disease progression for clinical trials. Moreover, both clinical trial design and clinical practice would benefit from methods that can position individuals along a common disease timeline to inform cohort selection criteria and potentially assist in prognosis, respectively.

Disease progression modeling addresses this problem using computational methods to reconstruct long-term trajectories from short-term data. There are numerous methods that handle longitudinal data (i.e., more than 1 measurement per individual), which have been applied to a wide range of neurologic diseases to reveal new clinically useful information (see reference 6 for a comprehensive review). The methods most directly relevant to our work are continuous models of biomarker dynamics, which have seen broad application in AD.^7-10^ These models are regression based and typically involve fitting parameters to data from multiple individuals to reconstruct trajectories of clinical^9^ and mixed biofluid and clinical and imaging markers.^7,8,10^ Notably, the authors in reference 9 introduced an individual-level time-shift into their regression model, allowing variability in the hidden individual-level disease time to be estimated directly from the data. More recently, Bayesian mixed-effects models with individual-level time-shifts have been developed.^11,12^ These models can make estimates of the shape and time scale of biomarker trajectories in progressive diseases, at the expense of requiring sigmoidal forms for the trajectories to be assumed a priori.

With respect to disease progression modeling in HD specifically, we previously trained an event-based model (EBM) to estimate the hidden sequence of sMRI changes using cross-sectional data from the TRACK-HD study.^13^ However, the EBM does not model longitudinal information, which is necessary to estimate the time between events and hence identify potential biomarkers and make individual-level prognoses. A continuous-time hidden Markov model was applied to longitudinal clinical test score data from the Enroll-HD study in HD.^14^ However, they did not perform any out-of-sample cross-validation and provided only limited examples of the model's predictive utility. Recently, the authors of reference 15 developed a generative model of individual- and group-level sMRI changes using a dynamic causal model (DCM). They found heterogeneous trajectories in regional volumes in a small (N = 49) gene-positive HD cohort from the TRACK-HD and TRACKOn-HD studies.^16,17^ However, the proposed DCM did not infer individual-level disease time, which necessitated large numbers of time points per individual to capture disease progression (an average of 5.94 time points per individual were used). This limits the possible cohorts that can be used to train the model, which by extension limits its broader applicability to unseen data, a key requirement for model validation and clinical utility. Furthermore, DCMs are based on parametric models and therefore require hypothesis testing to identify the functional form of each trajectory from the data. This typically limits trajectories to a symmetric form (e.g., sigmoidal), which is a reasonable assumption for brain volumes^18^ but precludes the possibility of a trajectory having more than one time-dependent rate of change (i.e., acceleration). Although not well reported, multiple accelerations could be biologically feasible; for example, if the rate of atrophy in a given brain region is changed by external factors (such as accumulation of proteinopathies^1^).

A nonparametric approach to modeling longitudinal changes is provided by Gaussian process (GP) regression, which allows one to capture temporal covariance without making limiting—and often unsuitable—a priori assumptions on the form of the distributions generating observed data.^19^ A GP model of normative individual-level changes in global gray matter abnormalities was proposed by the authors of reference 20. However, this model did not account for individual-level time-shifts and hence is not suitable for modeling disease processes. The GP progression model (GPPM) formulates GP regression within a disease progression modeling framework, allowing for simultaneous inference of group-level dynamics and individual-level time-shifts^21^; here, the authors also demonstrate the GPPM's capabilities in Alzheimer disease. The GPPM has 3 key strengths: (1) unlike the EBM in reference 13, it models temporal covariance, giving us information on the timescale of changes in HD; (2) unlike the DCM in reference 15, it infers individual-level disease time, allowing us to stitch together short-term measurements into long-term trajectories; and (3) unlike either EBM or DCM, it is nonparametric, allowing us to infer data-driven trajectories without making a priori assumptions. Furthermore, GPPM is generative, meaning that it provides probabilistic predictions and hence allows for direct quantification of prediction uncertainty.

Here, we apply the GPPM to learn a common disease timeline and hence model longitudinal trajectories of regional sMRI markers in HD. Next, we use the disease timeline of sMRI trajectories inferred by the GPPM to estimate, for the first time, the relative timescale and magnitude of regional volume changes across the HD brain. Finally, we also demonstrate that the GPPM can provide improved predictive utility of clinical onset over the current state of the art. We use the 2 largest imaging data sets in HD, TRACK-HD^16^ and PREDICT-HD,^22^ to train and test the GPPM, respectively. These data sets also allow us to test our model with unseen data from a completely separate study, providing a realistic measure of its clinical predictive utility, where individuals may be observed under completely different conditions (e.g., place, time) than those used to train the model. As such, we demonstrate the clinical validity and utility of our findings, which have broad implications for the use of sMRI markers in clinical trials and clinical practice.

## Methods

### Participants

We used T1-weighted 3T sMRI scans and genetic data (number of cytosine-adenine-guanine [CAG] repeats) from 284 participants (100 healthy control [HC]; 104 premanifest HD [PreHD]; and 80 manifest HD: HD) from the TRACK-HD study^16^ and 1.5 T and 3 T sMRI scans from 164 participants (36 HC [23 at 1.5 T; 13 at 3 T]; 128 [111 at 1.5 T; 17 at 3 T] PreHD) from the PREDICT-HD study,^22^ which corresponded to all available participants with measurements at 3 consecutive time points (baseline plus 2 follow-ups at 12-month intervals). Summary demographic data are shown in Table 1. We have previously studied these data sets in detail; see reference 23 for more detailed inclusion criteria, demographics, image acquisition protocols, and interstudy comparisons.

**Table 1:**
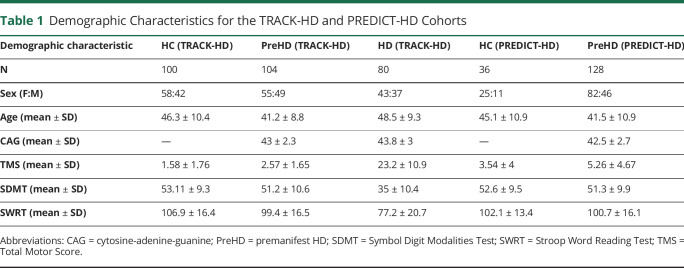
Demographic Characteristics for the TRACK-HD and PREDICT-HD Cohorts

### Standard Protocol Approvals, Registrations, and Patient Consents

The TRACK-HD and PREDICT-HD study protocols were approved by the local Research Ethics Committees, and participants signed consents for both participation and to allow deidentified research data to be sent to collaborative institutions for analysis.

### Image Analysis

We longitudinally registered scans to reduce intraindividual variability using the SPM12 tool,^24^ including differential bias correction for between timepoint scan inhomogeneities.^25^ Scans were then postprocessed using the Geodesic Information Flows segmentation tool^26^ to provide bilateral regional volume measurements. Rigorous manual quality control was performed on both the raw and processed images from every individual to remove noisy images and failed segmentations. All volumes were adjusted for covariates (age, sex, site, and total intracranial volume) by regressing against the HC group in each study separately. Field strength was also included as a covariate in the PREDICT-HD data set. We previously found consistent changes in regional volumes in the PreHD groups between studies.^23^

Ten adjusted regions of interest were then selected based on either clinical knowledge of HD pathology (caudate, putamen, pallidum, ventricles, thalamus proper, and sensory motor; see e.g., references 2, 27, 28) or to provide coverage of the 4 main regions of the cerebral cortex (frontal, temporal, occipital, and parietal). We trained GPPM using these volumes from gene-positive (PreHD and HD) individuals from TRACK-HD and tested GPPM using the PreHD group from PREDICT-HD. The CAG data are reserved for model comparison to a benchmark survival model (SM) that predicts time to onset using individual-level age and CAG repeat count.^29^

### Disease Progression Modeling

Longitudinal change in key regional volumes was modeled at both the individual and group levels using the GPPM introduced by the authors in reference 21. GPPM estimates a common timeline across the population, as well as a time-shift (position) for each individual along the timeline. Together, this information provides a staging system, with individual times given by the time-shift and prognosis given by the timeline.

More formally, GPPM implements time-reparameterized GP regression defined by the generative model:
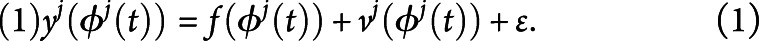


Here, 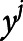 is the vector of regional volume measurements for subject *j*, 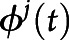
is the time reparameterization function for subject *j*, 
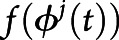
is the fixed-effect GP used to model group-level trajectories, 
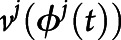
is the individual Gaussian random effect, and 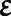 is time-independent measurement noise. The time reparameterization function 
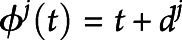
 defines the individual-level time-shift, 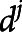, which is learned from the data. The model therefore allows for estimation of both longitudinal volumetric change at the group and individual levels and individual-level time-shift along the predicted trajectory. Furthermore, GPPM is formulated in a Bayesian framework, allowing estimation of the uncertainty over predicted trajectories. Monotonicity in the group-level volumetric evolution was enforced by requiring the first derivative of the fixed-effects function to be positive. Model parameters were estimated using the Deep GP variational framework presented by the authors in reference 30 and implemented in PyTorch (pytorch.org).

To define a common threshold for biomarker abnormality, we shifted trajectories such that the magnitude of each trajectory at t = 0 was equal to the mean volume of the biomarker for the manifest HD group at baseline. After the GPPM was trained, individual-level time-shifts in the test data were then estimated as the time at which the difference between the average fixed-effect trajectory and the individual regional volume measurements was minimized. As such, GPPM could be used in clinical practice to stage individuals using postprocessed sMRI data from a single observation. This staging could then be used to inform prognosis, based on their position along the regional brain volume trajectories.

### Data Availability

Anonymized data and documentation from this study can be made available to qualified investigators on reasonable request. Such arrangements are subject to standard data sharing agreements.

## Results

**Figure 1:**
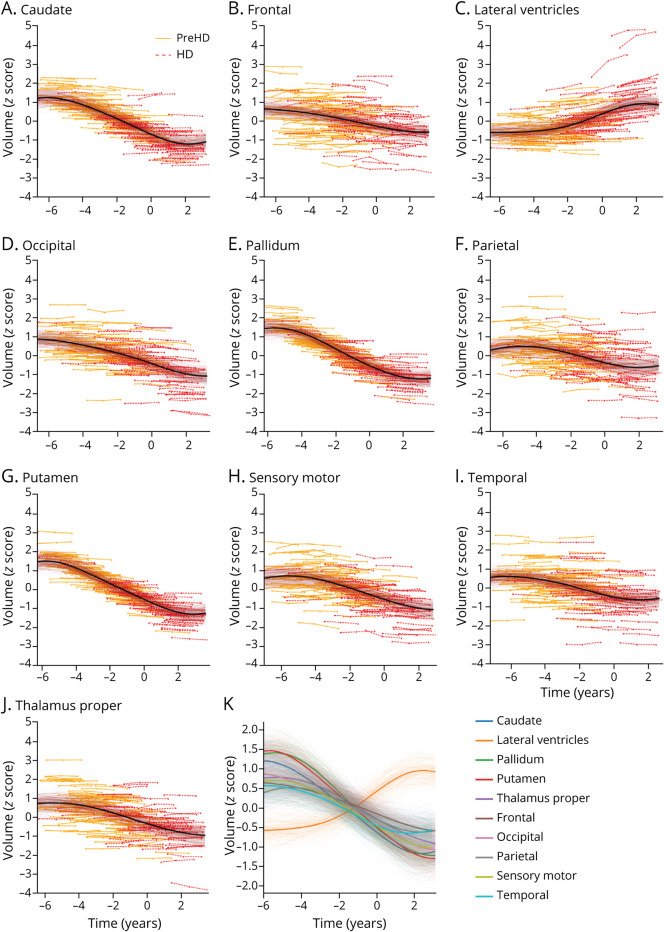
Regional Brain Volume Trajectories in the TRACK-HD Cohort Regional brain volume trajectories in the TRACK-HD cohort. (A-J) Individual regional volume trajectories, with PreHD individuals in solid orange lines, HD individuals in dashed red lines, GPPM average fit as a solid black line, and GPPM uncertainty as shaded red lines. (K) All regional volume trajectories overlaid. Standardized volumes (y-axis) are shown, and the time-scale (x-axis) is centered such that t = 0 when the fitted trajectory (black line) is equal to the mean value of the HD group. Uncertainty in the fit is shown as light shading about the mean and was estimated using 200 samples from the posterior. GPPM = Gaussian Process Progression Model; HD = manifest Huntington disease; PreHD = pre-manifest Huntington disease.

Figure 1 shows regional brain volume trajectories in 10 key anatomic regions inferred by GPPM in the TRACK-HD cohort. Uncertainty in the fitted models is obtained from 200 samples from the posterior. We assessed the quality of our trained model using 10-fold cross-validation, which returned a residual ratio 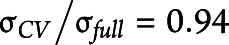, where 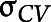
is the mean cross-validated residual, and 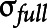
is the residual when fitting to the full data set, indicating high consistency of model hyperparameters. All regions show a monotonic change in volume over a period of ∼11 years (difference between the maximum and minimum individual-level times). Trajectories generally follow an approximately sigmoidal form, except the lateral ventricles that are initially flat before increasing rapidly ∼2 years before onset. We quantitatively assess the degree of nonlinearity for each trajectory by separately fitting a linear model to the transformed data and calculating the difference between this model and the GPPM trajectory (Table 2). We find that the pallidum has the highest degree of nonlinearity, followed by the lateral ventricles and sensory motor, with the frontal region demonstrating the most linear change.

**Table 2:**
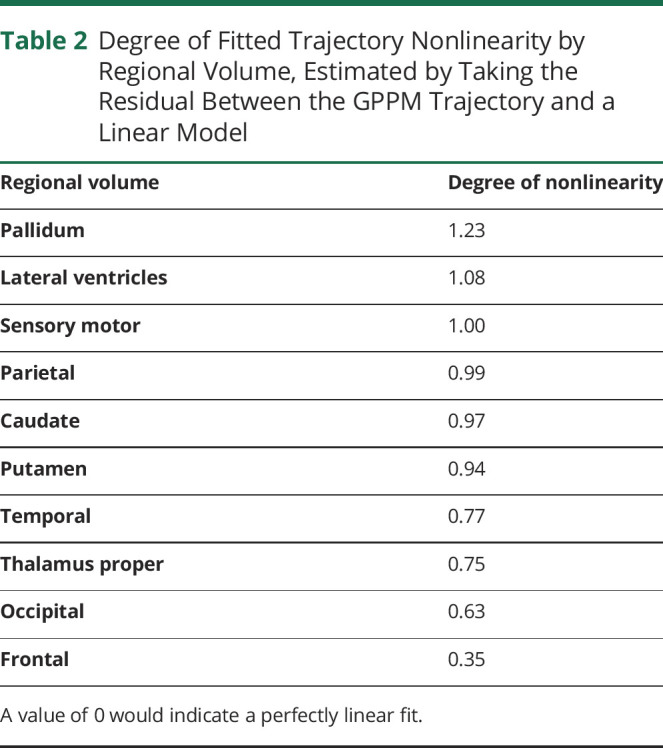
Degree of Fitted Trajectory Nonlinearity by Regional Volume, Estimated by Taking the Residual Between the GPPM Trajectory and a Linear Model

**Figure 2:**
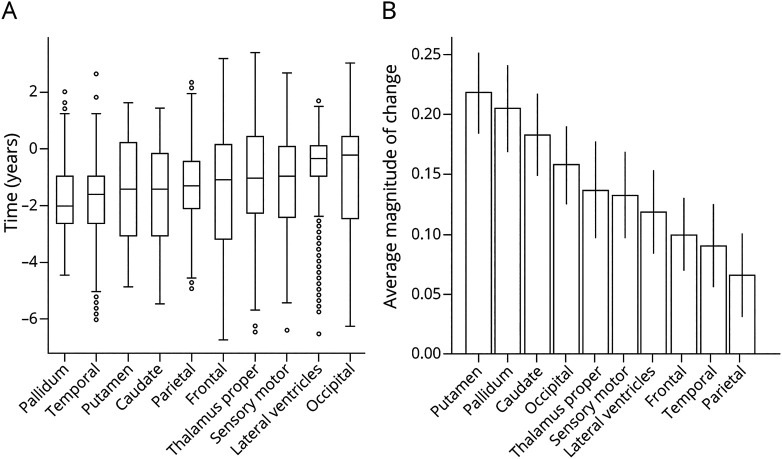
Time and Magnitude of Regional Brain Volume Trajectories in the TRACK-HD Cohort Time and magnitude of regional brain volume trajectories in the TRACK-HD cohort. (A) Predicted maximum change times for ten regional volumes from genotype-positive trajectories in TRACK-HD. Box-plots represent the mean, upper and lower bounds from 1,000 samples from the posterior. Extreme values are represented as black circles, while the green bars indicate the medians. (B) Average magnitude of change of each volume.

GPPM can also be used to estimate the most likely ordering of brain volume changes, by ordering volumes according to the time at which each trajectory reaches its maximum gradient (i.e., its change point). Figure 2A shows boxplots of the maximum change time for 10 regional volumes, inferred by drawing 1,000 samples from the posterior for each region, and Figure 2B shows the average magnitude of change for each region from the same set of samples.

**Figure 3:**
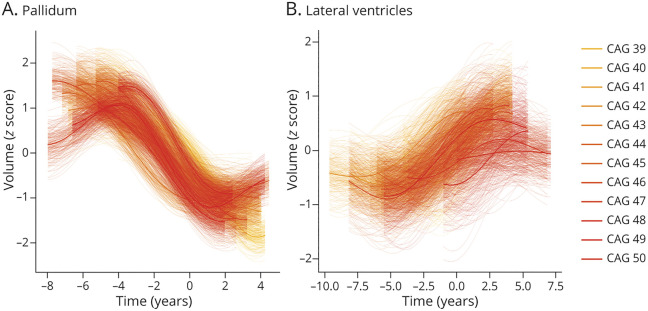
Selected Regional Brain Volume Trajectories by CAG Repeat Count in the TRACK-HD Cohort Selected regional brain volume trajectories inferred by GPPM from gene-positive (PreHD and HD) individuals from the TRACK-HD cohort, grouped by CAG repeat count. Standardized volumes (y-axis) are shown, and the time-scale (x-axis) is centered such that t = 0 when the fitted trajectory is equal to the mean value of the HD group. Uncertainty in the fit is shown as light shading about the mean and was estimated using 200 samples from the posterior. CAG = cytosine-adenine-guanine; GPPM = Gaussian Process Progression Model; HD = manifest Huntington disease; PreHD = pre-manifest Huntington disease.

To qualitatively investigate the relationship between regional volume changes and CAG repeat count, we trained GPPM on subsamples of individuals grouped by CAG repeat count in TRACK-HD. Figure 3 shows selected examples in the pallidum, which generally shows faster and more severe changes with increasing CAG repeat count, and the lateral ventricles, which does not exhibit any qualitative dependency. For the full set of regions, see eFigure 1 (links.lww.com/NXG/A472).

**Figure 4:**
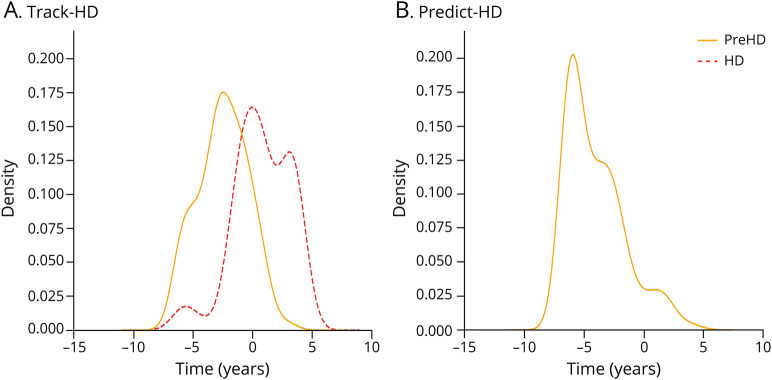
Group-Level Staging Density in the TRACK-HD and PREDICT-HD Cohorts (A) TRACK-HD and (B) PREDICT-HD. Distributions were fit using a nonparametric density function (KDE: kernel density estimate). HD = manifest Huntington disease; PreHD = pre-manifest Huntington disease.

To qualitatively evaluate the GPPM's within- and out-of-sample predictive utility, Figure 4A and B show the predicted disease time probability density distributions for gene-positive participants in the TRACK-HD and PREDICT-HD cohorts. Individuals were first assigned the most likely time averaged over all trajectories given their regional volume measurements, and the time distribution probability density was then calculated across the PreHD and HD groups using a nonparametric density function (kernel density estimate). For the TRACK-HD cohort, GPPM successfully clusters the PreHD and HD subgroups, placing the PreHD group midway along the trajectory and the manifest HD group at the end; in the PREDICT-HD cohort, GPPM times the PreHD group on average earlier than the TRACK-HD equivalent (TRACK-HD PreHD mean time = −2.5 years; PREDICT-HD PreHD mean time = −4 years).

**Figure 5:**
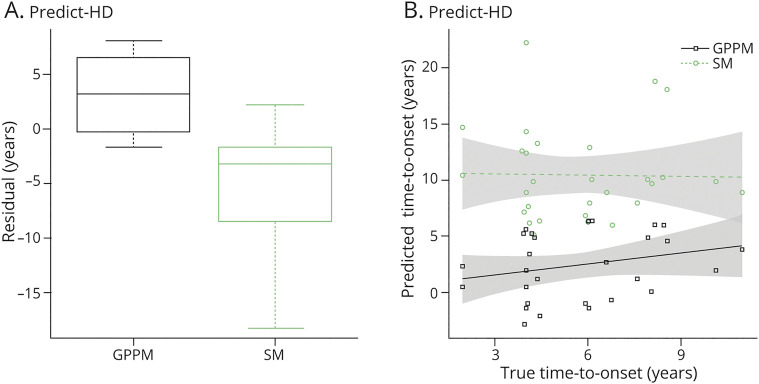
Predicted Time to Onset in the PREDICT-HD Cohort (A) Difference (residual) between actual and predicted time to onset for GPPM and SM, for PreHD individuals from PREDICT-HD. Boxplots show the median, first and third quartiles, and outliers. (B) Predicted and true time to onset for each model. Fits are from fixed effect linear models, with shaded bands corresponding to 95% confidence intervals. GPPM = Gaussian Process Progression Model; SM = survival model.

To quantitatively evaluate the GPPM's out-of-sample predictive utility with respect to the state of the art in prediction of onset, we compared GPPM with a benchmark nonparametric SM based on age and CAG repeat count.^29^ Figure 5 shows boxplots of the difference (residual) between the predicted and actual time to onset for each model. Here, 27 participants with PreHD at baseline from the PREDICT-HD cohort who converted to HD at any follow-up (not just within the 2 follow-ups used previously) are used to provide out-of-sample data. For the SM, hyperparameters obtained by the authors of reference 29 were used to parametrize the probability of time to onset given age and CAG repeat length, and 10^4^ samples were drawn from this distribution to obtain a mean predicted time to onset for each individual. For the GPPM, we defined the onset for each regional volume at t = 0, which corresponds to the mean volume for the manifest HD group.

The GPPM improves over the SM in terms of the absolute error, with the GPPM returning an absolute median residual of 2.9 years (±0.9, 5) and the SM 3.2 years (±1.2, 5.2) (see Figure 5A). However, the GPPM overestimated the time to onset (positive difference), whereas the SM underestimated (negative difference); overestimation is likely due to event censoring in the data (e.g., if individuals convert in-between visits), whereas underestimation is likely because of systematic model bias. Furthermore, the GPPM provides a smaller spread than SM; GPPM has root mean square error = 4.5 years and a maximum error of 7.9 years, whereas the SM has root mean square error = 6.6 years and a maximum error of 18.2 years. We further investigated the relationship between actual and predicted time to onset using linear regression; Figure 5B shows linear model fits for each model, with 95% confidence intervals. Although neither model shows significant coefficients, the GPPM does show a positive gradient between predicted and actual time to onset (beta = 0.3, *p* = 0.25), whereas the SM shows zero gradient (beta = −0.03, *p* = 0.93).

Finally, we note that we found no significant relationships under a fixed-effect linear regression of either actual or predicted time to onset and CAG repeat count, age, and their interaction in participants with PreHD from either cohort at baseline. A negative correlation between time to onset and CAG repeat count would be expected (see e.g., reference 31), but here significance in both the actual and predicted cases is precluded by the small sample size. We also investigated the relationship between predicted time to onset and 3 clinical variables^16^: Total Motor Score (TMS), Symbol Digit Modalities Test (SDMT), and Stroop Word Reading Test (SWRT). We found no significant associations between the model predictions and TMS (beta = −0.16, *p* = 0.16), SDMT (beta = 0.01, *p* = 0.8) or SWRT (beta = 0.05, *p* = 0.35), though all associations were in the expected directions (eFigure 2, links.lww.com/NXG/A472).

## Discussion

We have used GPPM to gain new data-driven insights into the spatiotemporal patterns of regional brain volume changes underlying HD progression. Our observations point to an informative method for selecting brain imaging markers for tracking disease progression; for example, we propose that the striatum (pallidum, caudate, and putamen) is most suitable for tracking acceleration earlier in HD progression, whereas the sensory motor and lateral ventricles can track acceleration at later times. As such, our approach has potential application in clinical trial design, where it could be used to identify the most suitable marker at the most suitable time to observe the effect of a given intervention. Moreover, we demonstrate that GPPM can be used to predict clinical onset of HD more accurately than the current state of the art. This supports the use of sMRI, when combined with GPPM, for potentially informing prognosis in clinical practice and stratification in the clinical trial design.

GPPM allows us to obtain data-driven trajectories of sMRI changes across the brain during the premanifest to manifest period in HD (Figure 1). We observe nonuniform atrophy across the brain (Figure 2B), with the largest changes (∼18%–22%) occurring in the striatum (caudate, pallidum, and putamen), and gradual change (∼7%–16%) across the 4 main regions of the brain (parietal, temporal, frontal, and occipital), over a period of ∼11 years. Our timescale agrees with previously reported observations of the timescale of sMRI changes in HD,^32^ where the authors reported that the rate of putamen and caudate atrophy becomes significant approximately 9 years and 11 years from estimated onset, respectively. However, the authors in reference 32 used direct regression methods applied to a small sample (N = 19) of hand-picked individuals who were observed over the whole period from premanifest to manifest HD. The GPPM sidesteps the need for tracking individuals across the premanifest to manifest period, which is costly (both in terms of financial and human resources) and potentially biased toward individuals who are willing and physically capable of being observed over many years.

Similarly, although the DCM used by the authors in reference 15 does not directly infer a timeline, the cohort they use spans 10 years from premanifest to manifest HD, over which they report ∼20% change in subcortical volumes (putamen and caudate), which agrees with our observations. However, we observe much larger changes in cortical regions; for example, in our analysis, the occipital region changes by 16% ± 3%, whereas the occipital regions reported by the authors in reference 15 change much less (occipital gyrus: 7% ± 1%, occipital pole: 5% ± 1%). This is most likely because our model includes substantially more manifest HD individuals; the authors in reference 15 include only PreHD at baseline, although all individuals underwent conversion to manifest HD over the period of observation. This highlights another advance of our analysis over previous analyses, as we can use GPPM to stitch together short-term longitudinal data from individuals with PreHD and manifest HD, allowing us to capture a broader picture of dynamic changes.

We note that the magnitude of change we observe in the striatum regions is larger than (approximately twice) that reported by the TRACK-HD investigators over the same period,^2^ Figure 2; compare differences between PreHD-A and HD2 trajectories. This is to be expected, as the results from the authors in reference 2 are for the mean of each diagnostic group, whereas GPPM effectively sorts individuals by disease time, therefore allowing us to measure change between early PreHD and late HD individuals.

As shown in Figure 2A, GPPM predicts the earliest changes in subcortical regions of the striatum (pallidum, putamen, and caudate), followed by cortical regions (temporal, frontal, and occipital), followed by the remaining regions (lateral ventricles, sensory motor, thalamus proper, and parietal lobe). These predictions largely agree with reported observations of early change in the striatum in HD^32-34^ and in particular using group-level analysis of voxel-based morphometry in the TRACK-HD cohort^16^ (although the authors of that study report earlier change in the occipital, which is likely because of variability in the signal from this region). Furthermore, we have also reported predictions of early change in the striatum using an EBM of regional brain volume changes in HD.^13^ Unlike our previous model, GPPM also provides the timescale of changes. In the TRACK-HD cohort, GPPM predicts the maximum rate of change in the pallidum and putamen ∼2 years before reaching the threshold for abnormality (as described in the Methods section, we define this threshold as the mean regional volume in the manifest HD group). These changes are followed by a rapid cascade in the remaining regions over a period of ∼1 year. We note that model inferences are limited by the training sample and that TRACK-HD was designed to preferentially include a cohort of PreHD individuals with a higher likelihood of converting during the study^16^; as such, the GPPM time estimates are likely shorter than would be expected from an average PreHD individual.

GPPM also allows us to observe varying degrees of nonlinearity in both cortical and subcortical regions (Table 2). Most subcortical regions (pallidum, caudate, putamen, and lateral ventricles) exhibit nonlinear behavior except the thalamus proper, and most cortical regions (parietal, temporal, occipital, and frontal) exhibit linear behavior except the sensory motor. Combined with the timescale of changes, this information has interesting implications for imaging marker selection for clinical trials. For example, caudate volume (which we previously identified as a strong candidate marker for phase 1 and 2 trials^26^) exhibits accelerated volume loss ∼5 years before average abnormality. This acceleration would need to be modeled when comparing treatment effects between groups, which motivates the use of nonlinear methods such as GPPM that can capture nonlinearity, unlike standard linear models.

A key genetic driver of HD progression is CAG repeat count, which has a well-reported correlation with the rate of clinical progression.^31^ With respect to sMRI volumetric change, the authors in reference 15 found that the CAG repeat length is related to the rate of cortical and striatal atrophy; this is a strength of their modeling approach, which accommodates individual-level covariates such as genetic burden. The association of brain volume loss on CAG repeat length in TRACK-HD and TRACKOn-HD was also reported by.^35^ Although GPPM does not (currently) explicitly account for individual-level covariates, we qualitatively investigated the effect of CAG repeat count by training models on subsamples grouped by CAG (Figure 3). In certain regions (e.g., the pallidum) our results suggest a dependency of CAG on the rate of volumetric change, whereas in other regions (e.g., the lateral ventricles), we did not observe any dependency. However, we caveat these observations with the fact that they are qualitative (e.g., no statistical hypothesis tests were performed) and that our sample sizes are limited (for example, the CAG = 50 group comprises N = 5 individuals; see eTable 1 [links.lww.com/NXG/A472] for the number in each group).

After confirming model quality using cross-validation, we used GPPM to infer individual-level disease times (Figure 4). GPPM was able to successfully stage the TRACK-HD PreHD group at a later stage than the PREDICT-HD PreHD group, which reflects our prior knowledge of differences between the 2 study cohorts; we have previously shown that the TRACK-HD PreHD group has a higher disease burden (product of age and CAG repeat length) than the PREDICT-HD PreHD group.^26^ We also evaluated GPPM with respect to clinical test score data, which showed nonsignificant dependencies on predicted time to onset, but in the expected directions.

We also demonstrated that GPPM can provide improved accuracy of prediction of onset than the current benchmark on out-of-sample data (Figure 5). Of interest, a similar trend was reported by the authors in reference 10 in Alzheimer disease, who found that their disease progression model overestimated and SM underestimated time to onset, respectively, and the disease progression model provided greater accuracy than the SM. This may be due to the exponential form of SMs, which allow for a longer tail of predictions further from onset (and would explain the asymmetric distributions observed in Figure 5A and presented by the authors of reference 10 in Figure 6B).

There are 3 main limitations of our analysis. First, we only analyze sMRI measurements, as the focus was on investigating regional brain volume changes in HD. However, GPPM can easily accommodate any dynamic marker—such as other imaging modalities, clinical test scores, and biofluids, as demonstrated by the authors of reference 21. Note that our use of an automated segmentation tool to obtain regional volumes must be considered when interpreting the biological relevance of our results. However, as noted by the authors of reference 26, we limited potential biases due to automated methods by performing rigorous manual quality control at every step of the imaging pipeline. Second, GPPM currently models only the individual-level time-shift, but not the individual-level rate of change. We are therefore unable to stratify individuals according to the rate of change in a given marker, which is of interest for the clinical trial design, where, for example, fast progressors might be excluded for a particular trial. However, although full Bayesian inference of the rate and time-shift is challenging because of the interaction between the 2 variables, it would be reasonably straightforward to add an additional variable in Equation 1 to account for individual-level random effects such as genetic factors; this is essentially the same approach used by the authors of reference 15. We propose possible extensions of GPPM such as this for future work. Finally, we are limited by the data sets used to train and test the model; the TRACK-HD cohort represents individuals who were likely to convert from PreHD to HD during the scope of the study, and the PREDICT-HD cohort used here entirely comprises PreHD individuals. To capture the full disease timeline, a younger cohort—such as the HD-YAS cohort^36^—would need to be included to cover the complete natural history of HD. We plan to combine HD-YAS with the TRACK-HD data set in future work, which will allow us to uncover the natural history of spatiotemporal sMRI changes across the preclinical HD timeline.

### Study Data and Code

Access to the TRACK-HD and PREDICT-HD data sets can be requested via the study leaders (SJT and JSP, respectively). The GPPM user interface is available at: gpprogressionmodel.inria.fr.

## Glossary

AD: Alzheimer disease
CAG: cytosine-adenine-guanine
DCM: dynamic causal model
EBM: event-based model
GP: Gaussian process
GPPM: GP progression model
HC: healthy control
HD: Huntington disease
PreHD: premanifest HD
SDMT: Symbol Digit Modalities Test
SM: survival model
sMRI: structural MRI
SWRT: Stroop Word Reading Test
TMS: Total Motor Score
